# Cranial Nerve Palsies and Peri-Mesencephalic Subarachnoid Haemorrhage: A Case Report and Review of the Literature

**DOI:** 10.7759/cureus.76043

**Published:** 2024-12-19

**Authors:** Susruta Manivannan, Vishnu A Suresh, Ardalan Zolnourian, Nicholas Borg, Jason Macdonald, Diederik Bulters

**Affiliations:** 1 Neurosurgery, Southampton General Hospital NHS Foundation Trust, Southampton, GBR; 2 Neuroradiology, Southampton General Hospital NHS Foundation Trust, Southampton, GBR

**Keywords:** bilateral oculomotor nerve palsy, cranial nerve palsy, perimesencephalic haemorrhage, subarachnoid haemorrhage, unilateral abducens nerve palsy

## Abstract

Peri-mesencephalic subarachnoid haemorrhage (PMSAH) is considered to be a clinically benign subset of subarachnoid haemorrhage (SAH). Cranial nerve palsies have been previously reported as rare sequelae of PMSAH. Herein, we report an unusual case of multiple cranial nerve palsies as a presenting feature of PMSAH and a review of the literature for cranial nerve palsies post-PMSAH. A 60-year-old gentleman was admitted with a sudden onset headache, bilateral oculomotor nerve, and unilateral abducens nerve palsies. CT demonstrated PMSAH, and angiography excluded a structural cause. On longer-term follow-up, he had persistent left-sided oculomotor nerve palsy. To the best of our knowledge, this is the first report of multiple cranial nerve palsies as a presenting feature of PMSAH and demonstrates the possibility of persistent neurological deficit following PMSAH.

## Introduction

Peri-mesencephalic subarachnoid haemorrhage (PMSAH) was initially identified in 1985 as a clinically benign subset of subarachnoid haemorrhage (SAH) [1]. PMSAH is defined as a haemorrhage within the basal cisterns anterior to the midbrain and pons with no identifiable causative vascular lesion on the digital subtraction angiogram (DSA) [2]. It typically presents with sudden-onset headaches, nausea, and vomiting. In contrast to SAH, however, deterioration in the level of consciousness or collapse is uncommon [3]. Additionally, the incidence of cranial nerve palsies in the context of SAH has previously been reported to be as high as 8%. In contrast, only a handful of reports in the literature describe this phenomenon occurring in PMSAH [4]. These include olfactory [5], oculomotor [6-9], trochlear [10], and bilateral abducens nerve palsies [11]. Here, we report the first case of bilateral oculomotor and unilateral abducens nerve palsies occurring in a single patient after PMSAH.

## Case presentation

**Figure 1:**
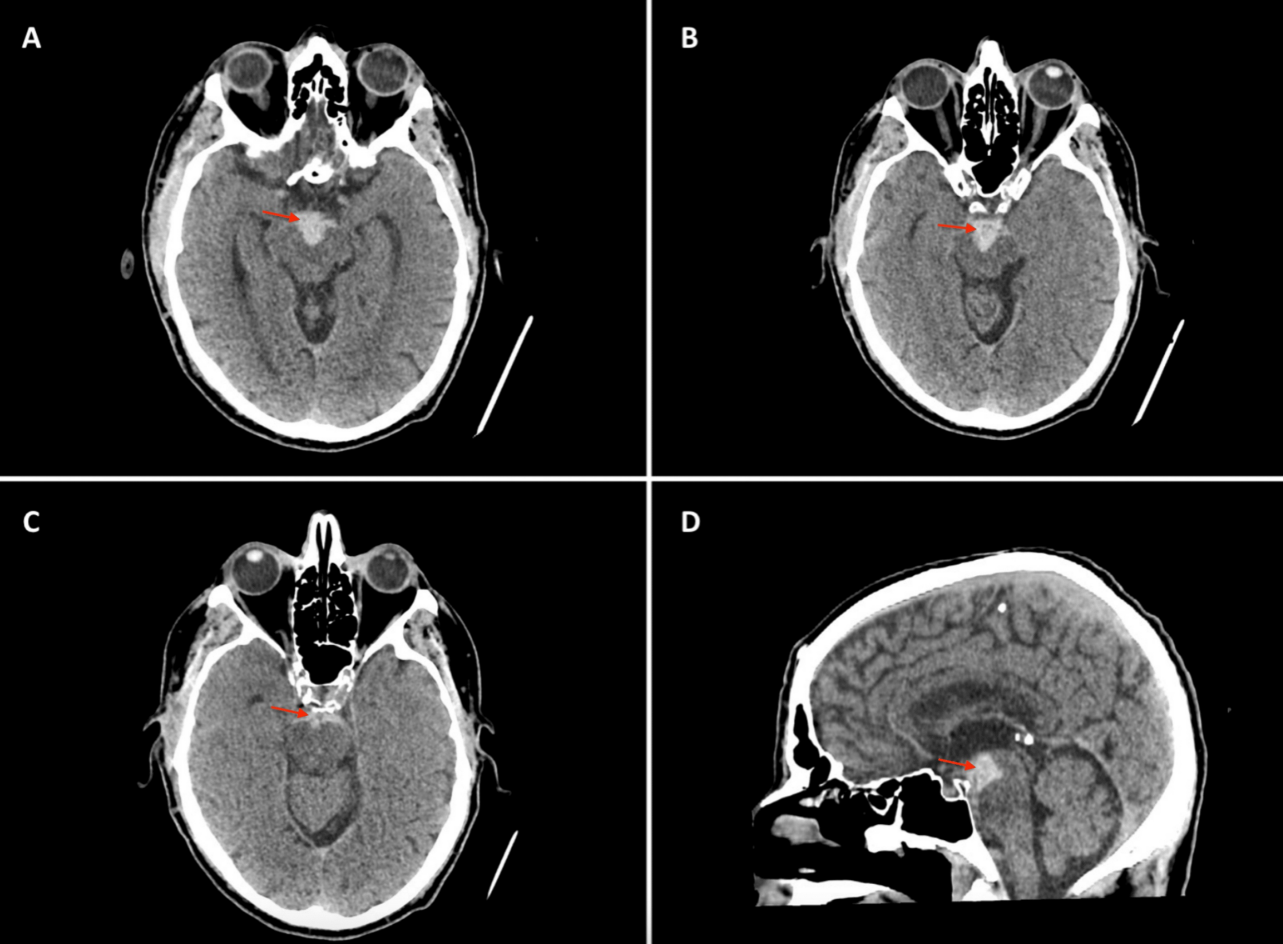
CT brain on initial admission. Arrows demonstrate localised acute subarachnoid haemorrhage within the pre-pontine and interpeduncular cisterns on axial (A-C) and sagittal (D) slices.

**Figure 2:**
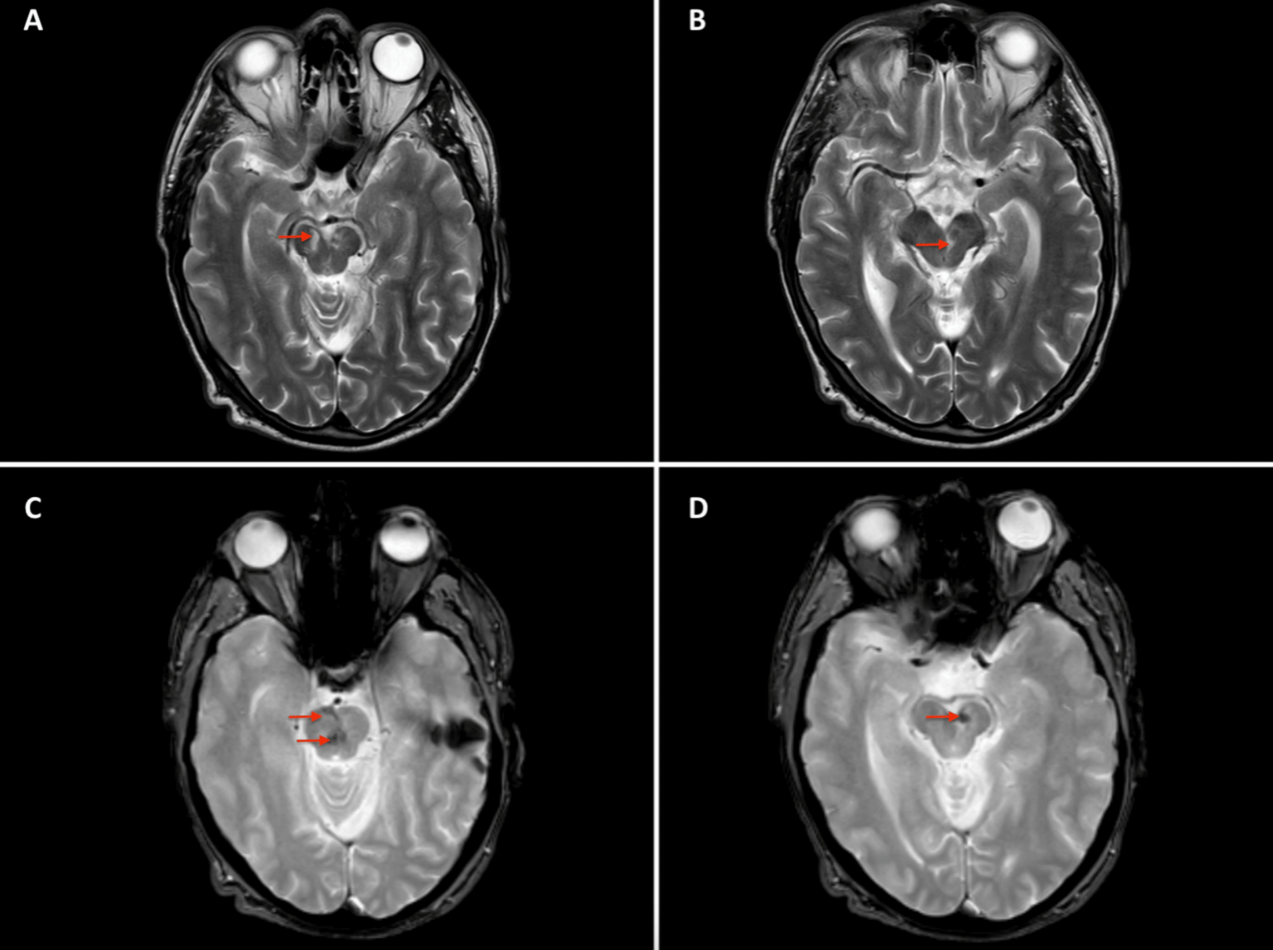
MRI brain at three month follow. Arrows demonstrate T2 hyperintensity within the midbrain and pons (A,B) and haemosiderin staining within and on the surface of the midbrain and pons, near the site of previous subarachnoid haemorrhage (C,D).

A 60-year-old right-handed male patient presented with a sudden-onset headache. His family subsequently found him drowsy with new gaze abnormalities. There were no reports of collapse, seizures, or nausea and vomiting. His past medical history included type 2 diabetes mellitus, hypertension, hypercholesterolaemia, and benign prostatic hypertrophy. On arrival at his local emergency department, he was reported to be confused and hypertensive, with mixed cranial nerve palsies and slurred speech. A non-contrast head CT in his local hospital showed SAH throughout the basal cisterns, mainly in the interpeduncular cistern with no extension into the proximal Sylvian or interhemispheric fissures (Figure 1). There was no evidence of an underlying aneurysm on the CT angiogram. Upon arrival at our unit, the patient's GCS was E1V4M6, indicating bilateral oculomotor nerve palsies (left complete, right partial, and pupil sparing), as well as a left abducens nerve palsy. The patient was commenced on a 21-day course of nimodipine for the prevention of vasospasm. DSA revealed no underlying vascular lesions. He was subsequently monitored for one week, during which he displayed ongoing cognitive difficulties and reduced mobility. An interval DSA at one week confirmed no underlying vascular lesions. He was transferred to his local neurorehabilitation unit and was discharged following one month of rehabilitation, during which his mobility improved. Follow-up MRI/MRA at three months demonstrated no evidence of underlying vascular lesion. Haemosiderin staining on T2-weighted MRI was noted within and on the surface of the midbrain and pons, close to the acute SAH seen in the pre-pontine cistern on initial presentation (Figure 2). At four months, the patient had resolution of both his right oculomotor nerve and left abducens nerve palsies, but his left oculomotor nerve palsy remained. Follow-up with the ophthalmology team at nine months post-PMSAH showed some improvement in his left oculomotor nerve palsy, but the patient still suffered from significant diplopia.

## Discussion

PMSAH is estimated to account for 6.8% of patients with spontaneous SAH [3], with a reduced risk of serious complications such as delayed cerebral ischaemia (DCI), hydrocephalus, and rebleed when compared with aneurysmal SAH [12]. A recent systematic review of PMSAH [3] examined presenting features, risk factors, and the clinical course of these patients. In contrast to its aneurysmal counterpart, a lower proportion of PMSAH patients were female, though the presenting age was similar at 53 years. Potential risk factors for PMSAH remain unclear.

Cranial nerve palsies are a rare feature of PMSAH. To date, reported cranial nerve palsies include olfactory, oculomotor, trochlear, abducens, and facial nerves (Table 1). Isolated unilateral oculomotor nerve palsies have been described previously in five cases [6-9]. The earliest case report described the course of a 49-year-old female with a delayed presentation of a painful oculomotor nerve palsy [6]. The initial presentation included a sudden-onset headache, visual disturbance, and neck stiffness. Interestingly, initial imaging demonstrated early evidence of hydrocephalus, which is relatively uncommon after PMSAH, and this was successfully managed with a lumbar drain. She was readmitted shortly after discharge with a rapidly progressive, painful oculomotor nerve palsy with pupillary involvement. A complete resolution was noted during the six-month follow-up. Given the evidence of distal basilar artery vasospasm on DSA and delayed presentation of neurological deficit, the authors hypothesised that cranial nerve palsy may have been secondary to vasospasm. Indeed, this is in contrast to subsequent case reports [7-9], which featured oculomotor nerve palsies on initial presentation and no evidence of vasospasm on imaging. These remaining four patients demonstrated similar features, including (i) sudden onset headache and oculomotor nerve palsy on preliminary assessment; (ii) no evidence of underlying vascular lesions on MRI, MRA, and DSA; and (iii) complete resolution of oculomotor nerve palsy at later follow-up.

**Table 1:** Studies reporting cranial nerve palsies in patients with perimesencephalic haemorrhage.

Study	Number of patients	Age, sex	Cranial nerve palsy	Time of onset	Outcome
Greebe et al. [5]	Nine	Age not stated; five were female	Olfactory	Unspecified	Transient in two patients; persistent in seven patients
Kamat et al. [6]	One	49, F	Oculomotor	2 days post-ictus	Resolved at six months
Reynolds et al. [7]	One	65, F	Oculomotor	Ictus	Resolved at six months
Sadamasa et al. [8]	Two	63, M	Oculomotor	Ictus	Resolved (unknown duration)
		66, M	Oculomotor	Ictus	Resolved at six weeks
Abbatemarco et al. [9]	One	63, F	Oculomotor	Ictus	Resolved at three days
Adachi et al. [10]	One	71, M	Trochlear	Ictus	Resolved at 16 days
Baldawa [11]	One	71, M	Abducens (bilateral)	1 day post-ictus	Died at five days
Wen et al. [13]	One	73, M	Facial	Ictus	Resolved at five days

A case series of 148 patients revealed anosmia in nine of them [5]. Of these, two experienced transient symptoms with resolution within three months, while the remainder had persistent symptoms at a mean follow-up duration of nine years. However, anosmia is a reported complication of intracranial surgery, aneurysmal SAH, aneurysmal clipping, and endovascular occlusion of intracranial aneurysms post-SAH [14-17]. Therefore, the authors concluded that blood within the basal cisterns must be a contributing factor to the development of cranial nerve deficit rather than raised intracranial pressure secondary to aneurysmal rupture or iatrogenic injury alone. Although the pathophysiology of PMSAH remains unclear, the general consensus is that it is likely secondary to venous rather than arterial bleeding, as is seen in aneurysmal SAH [18-20]. Given that a higher prevalence of anosmia has been reported in patients following endovascular treatment of ruptured aneurysms compared with unruptured aneurysms, it is likely that blood load is related to the risk of cranial nerve deficit [14,15]. This is supported by findings of cranial nerve deficits in patients with superficial siderosis of the central nervous system [21]. A recent case report also described transient unilateral left facial nerve palsy as a presenting feature of PMSAH [13], which resolved completely five days post-admission. However, forehead involvement was not reported, nor was the outcome of the MRI to rule out any evidence of underlying infarction.

There are several possible explanations for the development of cranial nerve palsies in PMSAH. Acute presentations are likely due to direct compression from the initial bleed or ischaemic injury to cranial nerves through compression of the intricate network of blood supply [22,23]. In cases of more delayed presentation, this may be secondary to irritation by blood and its breakdown products with possible vasospasm. This is supported by evidence of cranial nerve palsies in other pathologies involving blood degradation products within the subarachnoid space [21]. A case report of PMSAH presenting with left trochlear nerve palsy supports this explanation, given the specific distribution of subarachnoid blood within the left quadrigeminal cistern [10]. However, one may expect a right trochlear nerve palsy given the decussation of the trochlear nerves within the superior medullary velum prior to exiting the dorsal midbrain, suggesting that anatomical localisation based on imaging findings may not be entirely explanatory [24].

In our case, the lack of complete reversibility is the major difference when compared with other reported cases. We suspect that the MRI findings of T2 hyperintensity within the midbrain and pons (Figure 2) at the three-month follow-up are adequate explanations for this, which may support a possible ischaemic insult as a result of direct compression of the oculomotor nucleus or its blood supply. Acutely, evidence of blood within the pre-pontine and interpeduncular cisterns explains the involvement of both oculomotor and abducens nerves since: (i) the oculomotor nerve exits ventrally from the midbrain, medial to the cerebral peduncles, as a single root and passes through the interpeduncular cistern prior to crossing the petrous ridge of temporal bone and entering the cavernous sinus through the oculomotor porus [25]; (ii) the abducens nerve exits from the ventrolateral aspect of the ponto-medullary junction, running a steep course within the subarachnoid space along the clivus before piercing the dura to enter Dorello’s canal, providing a channel towards the cavernous sinus [26,27]. However, exceptions such as those involving a delayed presentation and suggestion of DCI [6] indicate the importance of ruling out potentially reversible causes. Our case, particularly with reference to the location of the blood on the diagnostic head CT (Figure 1), demonstrates how both oculomotor nerves were severely compressed against the cerebral peduncles and the midbrain. Therefore, direct pressure is more likely to be the cause of the nerve palsies in this case, as opposed to toxicity from the haemorrhage. The findings were also present at the time of presentation, which supports this theory. As the haemorrhage in the subarachnoid space was confined to the anterior midbrain and pons, it is likely that the abducens nerve was also compromised during its upward course over the clivus. As to why only one abducens nerve was affected, it is perhaps related to the asymmetrical anatomical course.

## Conclusions

Cranial nerve palsies are becoming an increasingly recognised feature of PMSAH. Possible mechanisms include direct cranial nerve compression by subarachnoid blood within the basal cisterns or ischaemic insult. In most cases, this is a short-term complication but can persist for several months in others. Although the oculomotor nerve is the most commonly affected, reports also indicate involvement of the olfactory, facial, trochlear, and abducens nerves. For the first time, we report a case of multiple cranial nerve palsies as a presenting feature of PSH.
